# Pros and cons of the health transformation program in Iran: evidence from financial outcomes at the household level

**DOI:** 10.4178/epih.e2017029

**Published:** 2017-07-18

**Authors:** Enayatollah Homaie Rad, Vahid Yazdi-Feyzabad, Shahrokh Yousefzadeh-Chabok, Abolhasan Afkar, Ahmad Naghibzadeh

**Affiliations:** 1 Social Determinants of Health Research Center, Guilan University of Medical Sciences, Rasht, Iran; 2 School of Management and Medical Informatics, Health Services Management Research Center, Institute for Futures Studies in Health, Kerman University of Medical Sciences, Kerman, Iran; 3 Guilan Road Trauma Research Center, Guilan University of Medical Sciences, Rasht, Iran; 4 Physiology Research Center, Institute of Neuropharmacology, Kerman University of Medical Sciences, Kerman, Iran

**Keywords:** Health care reform, Health expenditures, Health equity, Financial statements, Catastrophic illnesses, Iran’s health transformation program

## Abstract

**OBJECTIVES:**

The health transformation program was a recent reform in the health system of Iran that was implemented in early 2014. Some of the program’s important goals were to improve the equity of payments and to reduce out-of-pocket (OOP) payments and catastrophic health expenditures (CHE). In this study, these goals were evaluated using a before-and-after analysis.

**METHODS:**

Data on household income and expenditures in Guilan Province were gathered for the years 2013 and 2015. OOP payments for outpatient, inpatient, and drug services were calculated, and the results were compared using the propensity score matching technique after adjusting for confounding variables. Concentration indices and curves were added to quantify changes in inequity before and after the reform. The incidence of catastrophic expenditures was then calculated.

**RESULTS:**

Overall and outpatient service OOP payments increased by approximately 10 dollars, while for other types of services, no significant changes were found. Inequity and utilization of services did not change after the reform. However, a significant reduction was observed in CHE incidence (5.75 to 3.82%).

**CONCLUSIONS:**

The reform was successful in decreasing the incidence of CHE, but not in reducing the monetary amount of OOP payments or affecting the frequency of health service utilization.

## INTRODUCTION

In recent years, the Iranian health system has suffered from problems such as high out-of-pocket (OOP) spending and a higher than desirable rate of households faced with catastrophic health expenditures (CHE) [1]. Despite adopting several reforms, such as developing and implementing a rural family physician program, a rural insurance plan, and comprehensive national health insurance, there are still concerns about equitable and progressive financial protection and equitable access to health care [2]. For instance, a study on national health accounts showed that OOP spending increased from 40.63 to 54.64% between 2008 and 2014 [3,4].

These flaws compelled the policy makers of the Ministry of Health in partnership with cooperatives such as Labor and Social Welfare (formerly known as the Ministry of Welfare) to adopt a series of reforms known as the health transformation program (HTP). This program pursued several aims in a stepwise process, moving towards achieving universal health coverage. One of the most important objectives of the program was to reduce OOP expenditures, which are known to be the most inefficient and regressive form of financing mechanism [5,6].

To achieve its aims, policymakers implemented several interventions, including increasing the population covered by basic health insurance, reducing OOP expenditures for inpatient services, increasing the quality of care with a primary focus on the public hospitals affiliated with the Ministry of Health and Medical Education (MOH), and revising the medical tariffs to consider relative value units of clinical procedures as a way of establishing more balanced and realistic values for payments for different medical specialties [7]. This program also focused on improving the quality of services delivered in primary health care. This initiative concentrated on marginalized, slum-dwelling, and suburban populations (lacking access to preventive services) and on developing programs to control non-communicable diseases [8]. Allocating a proportion of the targeted subsidies initiative and value-added tax was determined to be the main financial source of support for the program. The first phase of this program, focusing on medical services and public hospitals of the MOH, was launched in May 2014, followed by a set of revised and updated medical tariffs in October 2014 [7,9].

Despite the presence of several challenges related to the HTP, which mainly arose from the opinions of influential stakeholders, there is little empirical evidence regarding the equity-oriented effects of this program. In order to evaluate the short-term effects of the HTP on equity in health care financing, we measured the treatment effect of HTP on OOP spending and households facing CHE in Guilan Province, a developed area located in the north of Iran [10]. In this province, which is located near the Caspian Sea, farming is the most important occupation of the rural population; additionally, it has the lowest fertility rate of the country [11,12].

## MATERIALS AND METHODS

### Data

In this study, data on household income and expenditures data from 2 years (2013 [before the reform] and 2015 [after the reform]) were compared. A household income and expenditure survey is conducted annually in all provinces of Iran to calculate economic indicators, such as the inflation rate. This survey is regularly performed by Iranian Statistical Center (ISC), a part of the Iran Management and Planning Organization, meaning that the authors did not have any conflicts of interest. In this survey, the monthly and annual overall expenditures of families for consumer goods before the survey were gathered. Using stratified methods for inhabitants of urban and rural regions for each province, households were selected as the study sample. The questionnaire used in this survey was based on Classification of Individual Consumption by Purpose [13]. All expenditures data were converted to US dollars after adjusting for the inflation rate. We did not use 2014 data, because the reform was implemented during this year and the effects of the reform were not clear. The dataset contained a total of 2,422 (2013: 1,205; 2015: 1,217) households living in both urban and rural regions of Guilan Province. Household OOP health expenditures for both inpatient and outpatient services were gathered by the ISC. Data on outpatient services and drug utilization were gathered over the course of one month, while data on inpatient services were gathered over the period of one year.

### Analysis

#### Before-and-after comparison of out-of-pocket and utilization

OOP health expenditures for inpatient, outpatient, and drug services were calculated for each household. A treatment effect estimator was used for comparing the OOP data before and after HTP. The treatment effect estimator, using the propensity score matching technique, matches 2 groups by an average of similar confounding covariates. In this method, mean confounding covariates are divided into blocks, and outcomes related to each block are compared. This method is often used for comparing national surveys with a large sample size. The potential confounding variables of this study were family income, the presence of children under 5 years of age, members of the family more than 70 years old, the number of illiterate people in the family, and the number of women in the family.

#### Before-and-after comparison of inequity in out-of-pocket expenditures and utilization

Concentration indices were calculated and a concentration curve was plotted to show the changes in relative inequality before and after the HTP. Concentration curves contain key variables: an outcome variable reflecting inequity and a variable capturing standards of living. In the present study, OOP payments and the utilization of health services for inpatient, outpatient, and drug services were considered to be outcome variables, and effective income was considered to reflect standards of living. Effective income was defined as the total consumption expenditures of household within a time period. Many studies have shown that effective income is a better than absolute income as indicator for living standards. A concentration curve plots the cumulative percentage of OOP expenditures against the income-ranked cumulative percentage of households (from the poorest to richest). Inequity in OOP expenditures (health services utilization) is assessed through comparison with a 45° line. If OOP payments (utilization) are higher for the poor, the concentration curve lies above the 45° line, and vice versa. The concentration index is the area between the concentration curve and the 45° line. It varies between -1 and +1, where negative values show higher payments or utilization among the poor and positive values show higher payments or utilization among the rich.

### Calculating catastrophic health expenditures

In this study, the Xu method was used to calculate CHE [14]. Households with health expenditures of more than 40% of their ability to pay them were grouped as households facing CHE. The ability-to-pay principle is based on the effective income after the deduction of subsistence expenditures. In this study, for calculating subsistence expenditures, the average per capita food consumption in quintiles of 45 to 55, after adjustment for household size (β= 0.56), was used. If the monthly household health expenditures were more than 40% of the ability to pay, a family was considered to have confronted CHE.

The present project received ethical approval from the Deputy of Research of Guilan University of Medical Sciences (approval no. 1197427392). The teeffect package was used for comparing 2 groups with a treatment effect estimator [15] and the conindex package was used for calculating concentration indices and comparing inequity in the groups [16]. All analyses were conducted using Stata SE version 13.1 (StatCorp., College Station, TX, USA).

## RESULTS

The average household size of the sample was 3.19 in 2013 and 2.99 in 2015. The mean age of each household in the study sample was 40.56 and 44.22 in 2013 and 2015, respectively. Furthermore, 45.02% of the households lived in urban regions in 2013. The corresponding figure was 44.81% in 2015.

### Comparing out-of-pocket expenditures and utilization

Tables 1 and 2 show the results of the descriptive analyses for years of 2013 and 2015. In Table 1, overall inpatient, outpatient, and drug OOP expenditures and the frequency of service utilization are shown. Overall OOP payments were 40.55 and 57.92 dollars before and after applying HTP in Guilan. Outpatient OOP payments were 31.34 dollars before the reform and 48.84 dollars after it. Before and after the reform, the annual inpatient OOP payments were 704.26 and 619.16 dollars, respectively.

In Table 2, the utilization of outpatient, inpatient, and drug services is shown. Before the HTP, outpatient, inpatient, and drug service utilization were 2.06, 0.27 and 0.83 times, while they were 2.08, 0.28, and 0.86 times after the reform, respectively.

Table 3 shows the results of comparing OOP expenditures and utilization before and after HTP using the propensity score matching technique. The outcome variables were adjusted by effective income, residency, household size, the number of males in household, the mean age of the household, and the number of literate people in the family. Overall OOP payments significantly increased after the reform (by 10.59 dollars). Outpatients OOP expenditures likewise showed a significant increase after the reform (by 10.94 dollars). OOP payments for inpatient and drug services exhibited no significant changes after the reform. In addition, the utilization of inpatient, outpatient, and drug services was not significantly different before and after the HTP.

### Comparing catastrophic health expenditures

Table 4 shows the percentage of households facing CHE before and after the reform. Before the reform, 5.75% of households faced CHE, while after the reform, 3.82% of families encountered CHE. Furthermore, as shown in Table 4, the percentage of households experiencing CHE was compared before and after the reform after adjusting for mean age, household size, effective income, residency, education, and number of males in the family. The adjusted rate of encountering CHE decreased significantly, by close to 2.39% (p= 0.02) after the reform.

### Comparing inequity

Table 5 shows the inequity in OOP expenditures and the utilization of overall, inpatient, outpatient, and drug services before and after the HTP. The p-values for comparing inequity before and after the reform were calculated for all estimations. The concentration index of overall OOP payments was 0.43 before the reform, and it decreased to 0.41 after it. However, this change was not significant. For outpatient OOP expenditures, the concentration index was 0.35, changing to 0.39 after the reform. The inpatient OOP concentration index was 0.69 and 0.50 before and after the reform, respectively. The drug services OOP concentration index was 0.26 before the reform, decreasing to 0.26 following the HTP. The outpatient utilization concentration index was 0.11 and 0.13 before and after the reform, respectively. The inpatient services utilization CI was 0.32 before the reform. After the reform, the figure declined to 0.25. For drug services utilization, the concentration index was 0.05 before the HTP, and after the reform it changed to 0.06. It is important to note that all findings regarding the concentration indices were statistically significant, but none of the changes in inequity before and after the reform were significant.

**Figure 1.**
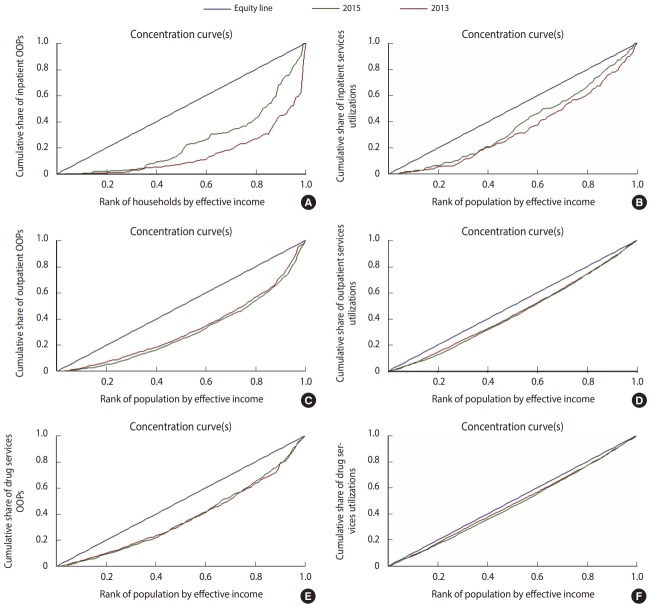
Concentration curves of inpatient (A, B), outpatient (C, D) and drug services (E, F) before and after the health transformation program. OOP, out-of-pocket.

Figure 1 shows the concentration curves of inpatient, outpatient, and drug services before and after the HTP. The red lines present the levels of inequity before the reform and the green line shows the corresponding after the reform. The closer the lines are to the equity line, the greater the equity.

Table 6 presents a comparison between the results of HTP with the health system goals. As shown in the Table 6, overall OOP payments and outpatient OOP payments increased significantly after the reform, which is a negative consequence of the reform. OOP expenditures for inpatient and drug services did not change after the reform. Moreover, the utilization of services showed no significant changes during this period. The number of households encountering CHE decreased significantly after the reform, in accordance with the aims of the project and health system. Inequity in OOP expenditures and utilization did not significantly change after the health reform.

## DISCUSSION

Following the implementation of the HTP, substantial increases in the overall and outpatient OOP payments of households were observed. However, no changes were observed in OOP expenditures for inpatient and drug services. The utilization of outpatient services declined after the reform, but for other types of services, no changes occurred. There was no change in inequity in either utilization or OOP expenditures during the reform. Similarly, in a survey done in the west of Iran, a reduction was found in OOP payments after the reform. However, in that study, the OOP payments were not divided by the type of services, but restricted only to services delivered in hospitals. In addition, the percentage of health expenditures decreased from 24% before the reform to 3% after the reform [6].

It is important to apprehend the difference between the monetary amount of OOP payments and the percentage of OOP payments. The aim of the HTP was to reduce the percentage of OOP payments for inpatient services to 10% of total health expenditures [7]. Much evidence has shown that the government was successful in achieving this goal. For example, in a study conducted in Sari, Iran, the authors reported that the percentage of OOP payments for drug and inpatient services decreased significantly. Moreover, a study in Isfahan reported that the percentage of OOP payments for inpatient services decreased to nearly 17% after the reform [17,18]. However, the present findings indicate that the monetary amount of OOP payments increased after the reform. Lower OOP payments are important when they increase financial access to health services for the population. Reducing the percentage of OOP payments may not be a good indicator of increased accessibility to health care services. It is important for policy makers to draw attention to the monetary amount of OOP payments. In Iran’s health reform, a major increase occurred in health staff payments as well as overall health expenditure, but the growth of OOP payments was not as high as that of overall and staff expenditures. Thus, the percentage of OOPs decreased and the monetary amount of OOPs increased simultaneously [5].

The frequency of utilization of health services and inequity therein did not change before and after the reform. One of the main goals of the HTP was to increase the accessibility to health care services for all. According to the results of the present study, it is neither the case that the poor utilized more than others nor that the rich did. Thus, the health care reform was not successful in increasing health care utilization. OOP payments did not change before and after the reform. Thus, the prices of health care services remained as high as before, and financial access to health care services did not change [19,20]. Inequity in OOP payments was also the same after the implementation of the HTP. Part of the HTP was extending the coverage of health insurance to include all people. Some reports have suggested that extending insurance coverage might lead to regressive health care financing and threaten equity in the financial aspects of health care [9].

Our findings showed that the rate of CHE decreased from 5.75 to 3.82%. Implementation of the reform was successful in decreasing the number of families facing a catastrophic situation. In a study performed in 2016 in the west of Iran, the researchers reported that the rate of CHE was high (4.89%). Although reducing the rate of CHE after implementing the HTP was discussed, no evidence of achieving the goals of the Fifth Development Plan of Iran was found (1%) [21]. Reducing the number of afflicted families was one of the main goals of the HTP. For this purpose, the MOH identified some chronic diseases as being catastrophic for families due to their high financial burden. It subsidizes the costs of these diseases for the poor. Identifying these diseases was easy for the MOH, and they could be easily separated from other condition. Thus, the MOH did indeed successfully implement its program.

This study had some limitations. Since we used data on household income and expenditures, no observations were available for the total health expenditures of each family, and we were not able to investigate changes in it. Therefore, the percentage of OOP payments could not be calculated. For future studies, it is suggested that the changes be tested in the percentage of OOP payments using a time series analysis.

This study aimed to financially evaluate the HTP in northern Iran. It discovered that the reform was not successful in decreasing OOP payments or the inequity of the payments, while it seems to have been successful in reducing the number of households experiencing CHE.

## Figures and Tables

**Table 1.** OOP payments for overall, outpatient, inpatient, and drug services before and after the health transformation program

OOP	Period	2013		2015	
		Mean	SE	Mean	SE
Overall	Monthly	40.55	81.32	57.92	89.33
Outpatient	Monthly	31.34	49.12	48.84	76.84
Inpatient	Yearly	704.26	127.18	619.16	70.14
Drug services	Monthly	13.07	0.74	18.14	0.83

Unit: dollars. OOP, out-of-pocket; SE, standard error.

**Table 2.** Frequency of utilization of outpatient, inpatient, and drug services before and after the health transformation program

Utilization	Period	2013		2015	
		Mean	SE	Mean	SE
Outpatient	Monthly	2.06	0.04	2.08	0.04
Inpatient	Yearly	0.27	0.02	0.28	0.02
Drug services	Monthly	0.83	0.01	0.86	0.01

SE, standard error.

**Table 3.** Comparison of OOP expenditures and utilization before and after the health transformation program after adjusting for confounding variables

Variable	Coefficient	SE	p-value	95% LLCI	95% HLCI
OOP					
Overall	10.59	3.65	0.004	3.42	17.75
Outpatient	10.94	2.64	<0.001	5.77	16.11
Inpatient	-4.12	28.13	0.88	-59.26	51.02
Drug services	1.47	1.26	0.24	-1.00	3.93
Utilization					
Outpatient	-0.04	0.07	0.51	-0.04	0.07
Inpatient	0.02	0.03	0.60	0.02	0.03
Drug services	0.02	0.02	0.28	0.02	0.02

OOP, out-of-pocket; SE, standard error; LLCI, lowest-likelihood confidence interval; HLCI, highest-likelihood confidence interval.

**Table 4.** Comparison of catastrophic health expenditures before and after the health transformation program

	Observations	Frequency	Prevalence(%)	SD	p-value
Before the reform	1217	70	5.75	0.23	
After the reform	1,205	46	3.82	0.19	
Adjusted difference	-	-	-2.39	0.01	0.02

SD, standard deviation.

**Table 5.** Comparison of inequity in OOP expenditures and utilization before and after the health transformation program

Variable	Before the reform		After the reform		Comparison	
	CI	SE	CI	SE	Difference	p-value
OOP						
Overall	0.43	0.05	0.41	0.04	-0.02	0.69
Outpatient	0.35	0.04	0.39	0.04	0.04	0.46
Inpatient	0.69	0.18	0.50	0.10	-0.19	0.35
Drug services	0.26	0.04	0.26	0.03	-0.00	0.96
Utilization						
Outpatient	0.11	0.01	0.13	0.01	0.01	0.40
Inpatient	0.32	0.05	0.25	0.04	-0.07	0.25
Drug services	0.05	0.01	0.06	0.01	0.01	0.25

OOP, out-of-pocket; CI, concentration index; SE, standard error.

**Table 6.** Comparison of the results of the health transformation program with the health system goals

Variable	Overall		Inequity	
	Results	Significant	Results	Significant
OOP				
Overall	Against	Yes	Against	No
Outpatient	Against	Yes	In agreement	No
Inpatient	In agreement	No	Against	No
Drug services	Against	No	Against	No
Utilization				
Outpatient	Against	No	Against	No
Inpatient	In agreement	No	In agreement	No
Drug services	In agreement	No	Against	No
CHE	In agreement	Yes	-	-

OOP, out-of-pocket; CHE, catastrophic health expenditures.
